# Effectiveness of advertising availability of prenatal ultrasound on uptake of antenatal care in rural Uganda: A cluster randomized trial

**DOI:** 10.1371/journal.pone.0175440

**Published:** 2017-04-12

**Authors:** William Cherniak, Geoffrey Anguyo, Christopher Meaney, Ling Yuan Kong, Isabelle Malhame, Romina Pace, Sumeet Sodhi, Michael Silverman

**Affiliations:** 1 Department of Family and Community Medicine, Division of Emergency Medicine, The Markham-Stouffville Hospital, University of Toronto, Toronto, Canada; 2 Bridge to Health Medical and Dental, Toronto, Canada; 3 Kigezi Healthcare Foundation, and Mbarara University of Science and Technology, Kabale, Uganda; 4 Department of Internal Medicine, McGill University, Montreal, Canada; 5 Department of Family and Community Medicine, Toronto Western Hospital, University Health Network, Toronto, Canada; 6 Division of Infectious Diseases, Western University, London, Canada; Centers for Disease Control and Prevention, UNITED STATES

## Abstract

In rural Uganda pregnant women often lack access to health services, do not attend antenatal care, and tend to utilize traditional healers/birth attendants. We hypothesized that receiving a message advertising that “you will be able to see your baby by ultrasound” would motivate rural Ugandan women who otherwise might use a traditional birth attendant to attend antenatal care, and that those women would subsequently be more satisfied with care. A cluster randomized trial was conducted across eight rural sub-counties in southwestern Uganda. Sub-counties were randomized to a control arm, with advertisement of antenatal care with no mention of portable obstetric ultrasound (four communities, n = 59), or an intervention arm, with advertisement of portable obstetric ultrasound. Advertisement of portable obstetric ultrasound was further divided into intervention A) word of mouth advertisement of portable obstetric ultrasound and antenatal care (one communitity, n = 16), B) radio advertisement of only antenatal care and word of mouth advertisement of antenatal care and portable obstetric ultrasound (one community, n = 7), or C) word of mouth + radio advertisement of both antenatal care and portable obstetric ultrasound (two communities, n = 75). The primary outcome was attendance to antenatal care. 159 women presented to antenatal care across eight sub-counties. The rate of attendance was 65.1 (per 1000 pregnant women, 95% CI 38.3–110.4) where portable obstetric ultrasound was advertised by radio and word of mouth, as compared to a rate of 11.1 (95% CI 6.1–20.1) in control communities (rate ratio 5.9, 95% CI 2.6–13.0, p<0.0001). Attendance was also improved in women who had previously seen a traditional healer (13.0, 95% CI 5.4–31.2) compared to control (1.5, 95% CI 0.5–5.0, rate ratio 8.7, 95% CI 2.0–38.1, p = 0.004). By advertising antenatal care and portable obstetric ultrasound by radio attendance was significantly improved. This study suggests that women can be motivated to attend antenatal care when offered the concrete incentive of seeing their baby.

## Introduction

Improving uptake of antenatal care services ie. getting more mothers to attend professional healthcare visits (using nurses, midwives or physicians) while pregnant is critical to improving maternal and child health.[1–4] Low uptake of antenatal care services (ANC) has been demonstrated in many low and middle-income countries.[5] Factors contributing to poor uptake include poor healthcare infrastructure,[6] parental education below primary school,[7,8] difficulty accessing healthcare,[7,9] reluctance to use modern medicine as opposed to traditional medicine,[10–12] and viewing ANC as a non-essential service.[3,12] Various methods have previously been trialed aiming to improve uptake of ANC services, such as utilizing community health networks,[13] initiating healthcare insurance,[14] and engaging male partners.[15] While these methods have demonstrated some success, attendance still remains sub-optimal in many areas. The benefit of delivering with a skilled birth attendant (SBA) or at a health care facility (HCF) versus homebirth have been clearly defined in the literature in rural regions across the world.[16,17] In Sub-Saharan Africa, it has been reported that the likelihood of use of a HCF for delivery increased 3.4 times after just one prenatal visit, and by nearly nine times after three ANC visits.[16]

Maternal-child care has been a pressing issue in Uganda in particular, with 360 maternal deaths for every 100,000 live births,[18] and one of the 20 highest under five national mortality rates in the world.[19] Amongst nations in Africa, Uganda has one of the highest population growth rates, with an annual increase of 3.2% and a fertility rate of 6.2%.[20] Additionally, it has been demonstrated that there is a large discrepancy in the proportion of women willing to deliver with a professional attendant between rural poor and urban poor (25% vs. 55%).[9] With approximately 1.5 million pregnancies per year, it is estimated that nearly 6000 women will die annually due to pregnancy related complications (16 mothers per day).[20]

Obstetric ultrasound imaging can help to decrease intrapartum complications as it permits early detection, diagnosis and intervention to prevent potential complications, while simultaneously improving trust in healthcare providers.[21–23] Obstetric ultrasound can indicate whether a mother is carrying a multiple pregnancy, has a breech presentation, a low lying placenta, or other complications. Equipped with this information, a mother and her partner can make an informed decision about whether or not to deliver at home or a health center where they can receive professional assistance[21–23] Additionally, studies conducted on the use of ultrasound technology in rural hospitals in Rwanda and Tanzania have indicated that after an initial training period, an ultrasound program led by local health care providers can be sustainable and lead to accurate diagnoses.[21,22] There has been recent discussion of utilizing portable (or compact) ultrasound in low and middle-income countries to bring healthcare to women who are isolated from standard health centers.[24]

To our knowledge, no published studies exist that have explored using portable ultrasound as an incentive to increase uptake of ANC services in rural disadvantaged populations. Therefore, we conducted a cluster-randomized controlled trial in rural communities in southwestern Uganda to assess the effectiveness of advertising portable obstetric ultrasound (pOBU) in increasing the rate of uptake of ANC services by pregnant women at the health facility (cluster) level. We hypothesized that advertisement of pOBU would increase the total number of women presenting to clinic. Of the pregnant women who do not use ANC services, some present to traditional healers/birth attendants (TBAs), whereas others do not seek out any assistance. As a secondary outcome, we also hypothesized that advertisement of pOBU would increase uptake of ANC services in the subset of women who would otherwise tend to seek care from a TBA.

## Material and methods

### Ethics approval and consent

Ethics approval was obtained for this study from Mbarara University of Science and Technology Ethics Board in Mbarara, Uganda as well as Lakeridge Health Ethics Board in Oshawa, Ontario (Canada). Approval was obtained on January 7^th^, 2014. All study participants were asked to provide written informed consent on a form developed in collaboration with the Mbarara University of Science and Technology (MUST) Ethics Board. Many study participants were illiterate or unable to read the consent form as it was written in English. A bilingual Ugandan midwife translated the consent form and verbally explained its contents for each study participant as they were checked into the clinic. It was clearly stated that any woman who chose to not engage in the study would still receive full ANC services.

### Study design

We conducted a non-blinded cluster-randomized controlled trial to evaluate the effectiveness of advertising prenatal pOBU on uptake of ANC services. This study design was selected to mitigate confounding variables and use the natural landscape of the region (described below). The trial was retrospectively registered at https://clinicaltrials.gov/ct2/show/NCT02587091 with registration number NCT02587091 and the original protocol is available at http://bridgetohealth.ca/study-protocol/. Retrospective registration was performed as our study was very low risk, and did not utilize any novel patient care. Rather, the standard of care was being provided to pregnant women in ANC clinics and the intervention was increasing access to care. There are no on-going trials and as such no further trial registries.

### Study population

The study was conducted from February 17^th^– 25^th^, 2014 in the Kabale District, a rural highland located in southwestern Uganda. It is roughly 560 km from the capital city of Kampala and has a population of approximately 522,000. It is divided into three regions—north, central and south, and is further subdivided into 22 sub-counties, with 11 in the north, four centrally and seven in the south (see S1 Fig).

The population of this region predominantly engages in subsistence agriculture and are similar from a cultural, language and ethnic background, often times known as Abanya-Kigezi or “people of Kigezi”. There is very limited data describing the healthcare of this rural and isolated region, and government statistics are likely inaccurate. The Ugandan Ministry of Health describes that healthcare provision is a mix of public health, provided on a district health system with level one to four health centers (four being the most advanced) and private healthcare, delivered predominantly by faith-based organizations such as the Uganda Catholic Medical Bureau (UCMB).[25] Uptake of any ANC services is close to 66%, with only one third of these women returning for a fourth visit or delivering in a HCF.[26]

All women who were currently aware of being pregnant and presented to ANC were eligible for inclusion into the study. There were no exclusion criteria for women in this study. Advertisement was carried out through a sequentially increasing intensity of word of mouth and/or radio messaging revolving around the central hypothesis.

### Randomization and masking

**Fig 1:**
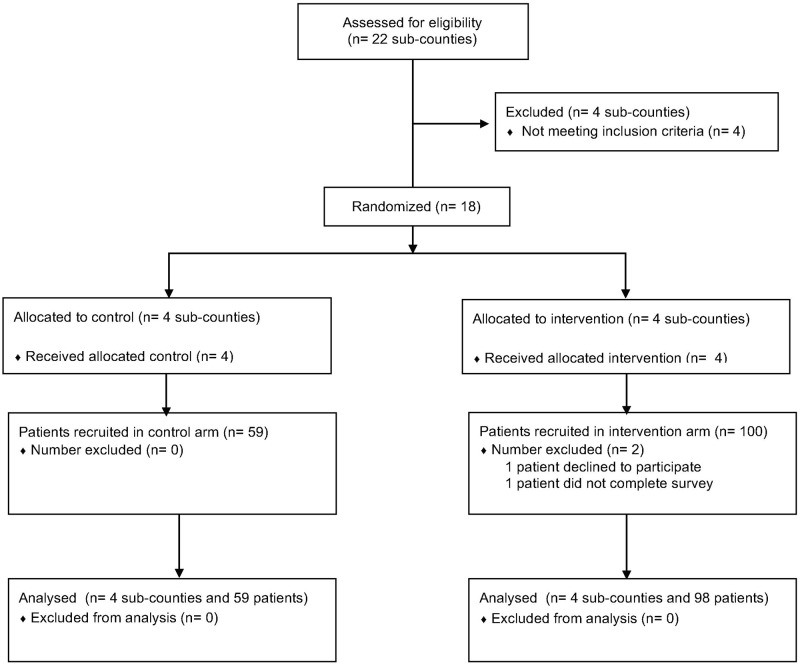
CONSORT flow chart.

Twenty-two sub-counties were assessed for inclusion. Four were intentionally excluded from the study design because they are primarily composed of urban and sub-urban communities and effectively divide the northern and southern regions. From the remaining 18 sub-counties four were randomly selected from the southern wing as the control arm (n = 4) and four from the northern wing as the intervention arm (n = 4) (Fig 1). Each sub-county represented one cluster. Selection of northern versus southern sub-counties for intervention or control arms, as well as selection of which sub-counties within each region and in which order they would be visited was all selected at random. Random selection was conducted by the statisticians at MUST using a simple random number program in MS-Excel.

The control sub-counties were chosen to be visited en bloc in the first four days, as opposed to alternating control then intervention on each day. This was done specifically to avoid contamination with messaging of pOBU, and was important in mitigating contamination with radio messaging in particular, which was felt to possibly cross geographic boundaries. Similarly the messages regarding pOBU were only delivered by radio on the last two days of the study in order to prevent contamination. Sunday was intentionally avoided as a study day as most study participants in the population ascribe to the Catholic faith and would not attend any clinics if held on that particular day.

When consent was obtained in the control arm there was intentional masking of pOBU. Following completion of initial consent and an entrance survey all women were provided with a debriefing form explaining the presence of ultrasound, including communities where it had not been advertised, and again consent was obtained to participate in the study. Any women who did not consent were provided with complete ANC services, including pOBU.

### Intervention

**Fig 2:**
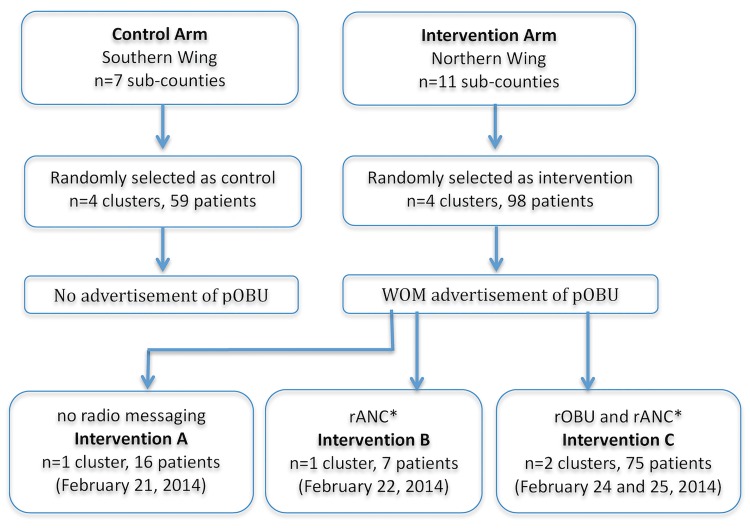
Forms of advertisement used for Antenatal Care (ANC) and portable Obstetric Ultrasound (pOBU) in control and intervention arms. WOM = word of mouth. rANC = radio advertisement of ANC, the radio message was, “there will be a free clinic for pregnant women at X community on X day, all tests and treatment will be provided free of charge”. rOBU = radio advertisement of portable obstetric ultrasound, the radio message was, “there will be a free clinic for pregnant women at X community on X day, all tests and treatment will be provided free of charge. Free ultrasound will also be offered at the clinic, if you come “you will be able to see a picture of your baby” and was delivered in both English and Ruchiga (the local dialect).

The intervention itself was advertising the availability pOBU at the cluster level (sub-county). The initial study design had only two arms as described above in randomization. However, the protocol was amended during the study due to interim analysis results demonstrating word of mouth (WOM) advertising of pOBU was not successful in increasing uptake of ANC. WOM advertising was carried out in the control arm by local community leaders and church pastors who announced the presence of the free ANC at community gatherings. In interventions A, B and C, ANC was advertised in the same way by WOM. In addition, a message regarding free pOBU was advertised by WOM. The message deliverd by WOM was not scripted for community leaders. They were given an introduction to the study and asked to tell their communities that pOBU would allow them to see a picture of their baby and that a free maternal health clinic would be offered along with the pOBU. It was hypothesized that many in the population—including community leaders—did not clearly understand what pOBU was, and therefore could not relay the message effectively. The intervention arm was subsequently modified to include radio advertising so that the message could be delivered more clearly and directly. Three nearly identical radio messages were delivered by the same Ugandan medical doctor through the same radio station over a four day period of time. The first radio message only discussed a free maternal health clinic, while the final two messages included a description of pOBU and the statement that “you will be able to see a picture of your baby”. This resulted in the following three distinct intervention arms: WOM advertisement by community leaders of both pOBU and ANC (Intervention A), WOM advertisement of pOBU and WOM + radio advertisement of ANC with no radio mention of pOBU (Intervention B), and WOM + radio advertisement of both pOBU and ANC (Intervention C). See Fig 2 for an outline of the intervention and control arms. The radio message in Intervention C included the words "You will be able to see a picture of your baby" delivered in both English and Ruchiga, the local dialect.

All women attending clinic were offered pOBU delivered by a certified Ugandan ultrasound technician. Study participants rotated through an ANC clinic that was based on the WHO guidelines for high quality ANC[5] as well as the WHO four-pronged approach of elimination of mother to child transmission of HIV (EMTCT).[27] S2 Fig depicts study participant flow in detail through the clinic. The ANC clinic was held alongside a mobile medical and dental health program which served as the site of the trial on each unique clinic day, with each day representing a clinic serving a unique sub-county. The clinics were coordinated and implemented by Bridge to Health Medical and Dental, a registered Canadian charity and humanitarian organization, and Kigezi Healthcare Foundation, a Ugandan not for profit community development organization.

Women received pre- and post-test counseling and rapid testing for HIV. Those who were positive were enrolled into the National HIV program and started on antiretroviral therapy as per national guidelines. Women were also offered intermittent presumptive therapy for malaria, rapid treponemal testing and treatment for syphilis, hepatitis B screening and syndromic management of sexually transmitted infections. Iron and folate supplementation was provided to all women. Any woman in active labour or with a critically low hemoglobin was transported to hospital by ambulance. Any woman with abnormal findings on ultrasound was referred for obstetric specialty follow up. All women were encouraged to deliver at a local HCF. All care was provided free of charge, with costs covered by Bridge to Health Medical and Dental.

### Survey

Women were interviewed on arrival and when leaving clinic using a standardized electronic entrance and exit survey (S1 File). This interview was performed with the assistance of a bilingual Ugandan midwife who translated the pre-developed survey into the local dialect, then translated responses into English. The entrance survey took approximately thirty minutes to complete and the exit survey approximately 10 minutes.

### Statistical analysis

Sub-counties were chosen as the unit of allocation and number of women accessing ANC in each sub-county as the unit of analysis.

The primary outcome was attendance to ANC between the intervention arm and control arm. Secondary outcomes included A) the attendance by women who had previously used a TBA, B) the attendance by women who had not yet attended ANC, C) the attendance between the three interventions and D) the number of women stating they came for pOBU.

The sample size calculation performed a priori was not utilized for this study as the design changed during implementation for reasons specified previously. We performed a post-hoc power calculation. We assumed that the distribution of pregnant women attending ANC was Poisson distributed. We assumed that the number of pregnant women in each sub-county was equal. We assumed that over-dispersion is not present when conducting the power calculation. Our goal was to test whether the rate of women attending ANC was equal in the control and intervention arms. Assuming a rate ratio of 2, we required 25 women attending ANC in the control arm and 50 women attending ANC in the intervention arm to achieve 80% power to reject the null hypothesis of equal rates of attendance.

Demographic data was summarized using simple descriptive statistics. We computed rates of ANC uptake, first ANC use and ANC uptake due to pOBU, respectively, and all at the cluster level. The count of the number of women attending ANC, for each specific outcome, formed the numerator of the rate. We used the number of women attending first ANC in 2013–2014 through government run clinics as our denominator. The data used in our denominator calculation was provided by the District Health Office through the District HMIS database. DHS and recent 2014 national census data only present bulk district data, and do not differentiate between subcounties. First ANC visit was chosen as the denominator as it represents the total number of recorded pregnancies in each sub-county over a specific and equal period of time. The ideal denominator would have been the total number of pregnant women in each community during the time period of each clinic day. This information was not available as sub-county lines were changed following the most recent census, and sub-county level data were not reported in the 2014 census. We expressed all rates per 1,000 women. Using Poisson regression, and Pearson’s correction factor for over-dispersion, we tested whether the ratio of rates in the control and intervention arms were equal.

Following our initial study design, we computed the results of the control arm versus all intervention arms combined together. We then computed all possible pair-wise rate ratios between the individual trial arms and the control arm. We chose to reject the null hypothesis of equal rates of ANC uptake at a 5% alpha level. When considering pair-wise comparison we apply the Bonferroni correction procedure, adjusting the statistical significance threshold by the number of pair-wise comparison conducted within a given model (six in our design). In this study design, this results in a Bonferroni corrected alpha level of: 0.05/6 = 0.0083. All statistical analyses were conducted using SAS version 9.4 (Cary, North Carolina). Since the outcomes reported were at the health facility (cluster) level, no further special analyses to account for clustering were conducted as per CONSORT statement guidelines.

Qualitative answers recorded in the exit survey were reviewed by a single analyst. Specific words pertinent to the context of the study (i.e., the idea of the ultrasound itself) were identified. Descriptors considered to be positive and negative were recorded.

## Results

In total, 59 study participants were recruited into the control arm and 100 study participants into the intervention arms. Out of these 159 study participants 100% initially consented to engage in the study. One out of 159 (0.6%) declined to continue after being informed of the ultrasound through the debriefing form, and 1/159 (0.6%) failed to complete the basic details of the entrance survey.

Study participants presenting to clinic across sub-counties were roughly similar according to their demographics and clinical backgrounds (Table 1). Specficially, most study participants were married at the time of the study, had similar maternal age, past pregnancies, education levels and past use of healthcare facilities (Table 1). Additionally, the religious composition and utilization of traditional medicine was similar between arms (Table 1).

**Table 1:** Demographic and clinical characteristics of women from the Uganda pOBU trial in the overall sample and in each of the intervention arms.

		Control	Intervention A	Intervention B	Intervention C
	Overall (N = 157)	Days 1–4 ANC = WOM (N = 59)	Day 5 ANC = WOM pOBU = WOM (N = 16)	Day 6 ANC = (WOM+radio) pOBU = WOM (N = 7)	Day 7–8 ANC = (WOM+radio) pOBU = (WOM+radio) (N = 75)
Age (years)–mean/SD	26.3 (5.6)	25.7 (5.5)	25.3 (5.4)	25.0 (7.0)	27.2 (5.6)
Education - Less than primary school - Primary/secondary school - Some/completed post-secondary	40 (25.5%) 99 (63.0%) 18 (11.5%)	15 (25.4%) 41 (69.5%) 3 (5.1%)	3 (18.8%) 9 (56.3%) 5 (24.9%)	3 (42.9%) 3 (42.9%) 1 (14.2%)	19 (25.3%) 46 (61.3%) 10 (13.4%)
Occupation - Subsistence farmer - Housewife - Other	106 (67.5%) 22 (14.0%) 29 (18.5%)	44 (74.6%) 12 (20.3%) 3 (5.1%)	14 (87.4%) 0 (0%) 2 (12.6%)	2 (28.6%) 2 (28.6%) 2 (42.8%)	46 (61.3%) 8 (10.7%) 21 (28%)
Religion - Catholic - Pentacostal - Protestant	104 (66.2%) 3 (1.9%) 50 (31.9%)	43 (72.9%) 0 (0%) 16 (27.1%)	12 (75.0%) 1 (6.3%) 3 (18.7%)	5 (71.4%) 0 (0%) 2 (28.6%)	44 (58.7%) 2 (2.7%) 29 (38.6%)
Relationship Status - Married - Not married	153 (97.5%) 4 (2.5%)	57 (96.6%) 2 (3.4%)	15 (93.8%) 1 (6.2%)	6 (85.7%) 1 (14.3%)	75 (100%) 0 (0%)
Ever had an Obstetric Ultrasound - Yes	27 (17.2%)	5 (8.5%)	7 (43.8%)	2 (28.6%)	13 (17.3%)
Ever Seen Traditional Healer for Health Care - Yes	26 (16.8%)	8 (16.6%)	1 (6.3%)	2 (28.6%)	15 (20.1%)
Number previous partners - 0 - 1 - 2+	119 (76.8%) 22 (14.2%) 14 (9.0%)	55 (93.2%) 3 (5.1%) 1 (1.7%)	11 (68.8%) 2 (12.5%) 3 (18.7%)	5 (83.3%) 0 (0%) 1 (16.7%)	48 (64.9%) 17 (23.0%) 9 (12.1%)
Number pregnancies - 1 - 2–3 - 4+	34 (21.8%) 59 (37.8%) 63 (40.4%)	13 (22.0%) 22 (37.3%) 24 (40.7%)	4 (25.0%) 9 (56.3%) 3 (18.7%)	3 (42.9%) 3 (42.9%) 1 (14.2%)	14 (18.9%) 25 (33.8%) 35 (47.3%)
Number previous live births - 0 - 1–2 - 3+	39 (25.7%) 65 (42.8%) 48 (31.6%)	14 (24.1%) 25 (43.1%) 19 (32.8%)	6 (37.5%) 7 (43.8%) 3 (18.7%)	3 (42.9%) 3 (42.9%) 1 (14.2%)	16 (22.5%) 30 (42.3%) 25 (35.2%)
Ever Had Home Delivery* - Yes	60 (48.8%)	26 (56.5%)	6 (50.0%)	3 (75.0%)	25 (40.1%)

ANC = Antenatal care. WOM = Word of mouth. pOBU = Portable ultra sound.

* Adjusted for number of primiparas

Following our initial study design, we first computed the results of all intervention arms combined together (A+B+C) versus the control arm (see Tables 2 and 3). This demonstrated that the rate ratio (RR) of attendance in the combined intervention arms (A+B+C) was greater than that in the control arm RR 2.69, (95% CI 1.06–6.81), P = 0.038. Rate of first ANC attendance and number seeing a traditional healer were not significantly different when comparing the combined intervention arms with the control arms. However, the RR of women attending clinic because they wanted to have a pOBU was significantly higher in the combined intervention arms (A+B+C) than in the controls RR 85.60, 95% CI 5.15–1422.49, P = 0.0019.

**Table 2:** Rates of Antenatal Clinic (ANC) attendance, attending an ANC clinic for the first time in current pregnancy, attending ANC after having seen a traditional healer/birth attendant, and attending ANC because of pOBU.

	Control				Intervention A	Intervention B	Intervention C	
	Days 1–4 ANC = WOM				Day 5 ANC = WOM pOBU = WOM	Day 6 ANC = (WOM+radio) pOBU = WOM	Day 7–8 ANC = (WOM+radio) pOBU = (WOM+radio)	
Clinic/village name	Hamurwa	Ruhija	Ikumba	Muko	Katanga	Kyanamira	Nyakigugwe	Mwisi
Number women attending first ANC with government (2013–2014)	1701	628	1058	1930	692	398	499	654
Number attending	14	15	16	14	16	7	44	31
Rate attendance in clinic	8.2	23.9	15.1	7.3	23.1	17.6	88.2	47.4
Rate attendance in clinic	11.1 (6.1, 20.1)				23.1 (7.4, 72.7)	17.6 (3.1, 99.4)	65.1 (38.3, 110.4)	
Combined Rate attendance in clinic	11.1 (6.1, 20.1)				29.8 (14.6, 60.9)			
Number first ANC	1	5	10	5	4	1	11	5
Rate first ANC attendance in clinic	0.6	7.9	9.5	2.6	5.8	2.5	22.0	7.6
Rate of first ANC attendance clinic	4.0 (1.5, 10.4)				5.8 (0.6, 53.6)	2.5 (0.03, 216.3)	13.9 (0.5, 42.3)	
Combined Rate of first ANC attendance clinic	4.0 (1.5, 10.4)				5.9 (1.1–32.1)			
Number seeing traditional healer	2	4	0	2	1	2	7	8
Rate seeing traditional healer	1.2	6.4	0	1.6	1.4	5.0	14.0	12.2
Rate seeing traditional healer	1.5 (0.5, 5.0)				1.4 (0.05, 42.7)	5.0 (0.5, 55.1)	13.0 (5.4, 31.2)	
Combined Rate seeing traditional healer	1.5 (0.5, 5.0)				4.6 (1.1, 18.7)			
Number stating they came to clinic because of pOBU	0	0	1	0	5	5	30	23
Rate stating they came to clinic because of pOBU	0	0	0.9	0	7.2	12.6	60.1	35.2
Rate stating they came to clinic because of pOBU	0.2 (0.01, 2.9)				7.2 (2.1, 24.7)	12.6 (3.7, 42.9)	46.0 (31.5, 67.0)	
Combined Rate stating they came to clinic because of pOBU	0.2 (0.01, 2.9)				16.1 (8.9, 29.1)			

ANC = Antenatal care. WOM = Word of Mouth. pOBU = Portable ultrasound. All rates expressed per 1000 women attending first ANC with government (2013–2014). The numerator of the rate is the count of the specific outcome under consideration; whereas, the denominator is the number of women attending first ANC with government (2013–2014). Rate calculations and 95% confidence intervals about the rates displayed for each of the control/intervention arms are derived from a fitted Poisson regression model (using Pearson’s scale correction factor to account for over-dispersion).

**Table 3:** Rate ratios of primary and secondary outcomes between arms.

Arm of Trial	Primary Outcome	Secondary Outcomes		
	Rate Ratio Attending	Rate Ratio Attending First ANC	Rate Ratio Seeing Traditional Healer	Rate Ratio Stating they Came for POBU
A, B and C versus Control	2.69 (1.06, 6.81) P = 0.038	1.48 (0.21, 10.53) P = 0.6927	3.03 (0.47, 19.29) P = 0.2412	85.60 (5.15, 1422.49) *P = 0.0019
A versus Control	2.08 (0.57, 7.58) P = 0.2653	1.46 (0.13, 16.63) P = 0.7588	0.96 (0.03, 34.88) P = 0.9824	38.42 (1.89, 779.01) P = 0.0175
B versus Control	1.59 (0.25, 2.29) P = 0.6221	0.64 (0.01, 60.81) P = 0.8459	3.34 (0.23, 48.59) P = 0.3774	66.80 (3.29, 1354.46) *P = 0.0062
C versus Control	5.86 (2.64, 13.01) *P<0.0001	3.51 (0.80, 15.41) P = 0.0957	8.65 (1.96, 38.09) *P = 0.0044	244.41 (15.27, 3912.46) *P = 0.0001
B versus A	0.76 (0.10, 6.06) P = 0.7963	0.43 (0.01, 63.30) P = 0.7430	3.48 (0.05, 220.13) P = 0.5559	1.74 (0.31, 9.88) P = 0.5327
C versus A	2.81 (0.80, 9.94) P = 0.1081	2.40 (0.20, 28.97) P = 0.4907	9.00 (0.27, 297.48) P = 0.2182	6.36 (1.76, 23.00) *P = 0.0048
C versus B	3.70 (0.60, 22.62) P = 0.16	5.52 (0.06, 545.22) P = 0.4658	2.59 (0.20, 33.14) P = 0.4646	3.66 (1.01, 13.23) P = 0.0479

A = Intervention A. B = Intervention B. C = Intervention C.

*statistically significant at Bonferroni corrected α/6 = 0.0083 level.

Comparing the various intervention arms separately we found that the attendance rate was significantly greater when pOBU and ANC were advertised by radio and WOM (intervention C) at 65.1 as compared to control communities with a rate ratio (RR) of 5.9 (Tables 2 and 3). The addition of advertising pOBU by word of mouth (Intervention A) or advertising the ANC by radio but pOBU by WOM (Intervention B) both failed to significantly increase ANC attendance compared with controls (Table 3). The rate of women who attended clinic who had seen a TBA was also significantly greater in intervention C as compared to control communities (Table 2). 42 out of 157 women (26.8%) had not been to ANC during their current pregnancy (Table 2). There was a nonsignificant trend towards a greater rate of first time attendance to ANC in intervention C compared to controls (Table 2). As shown in Table 3, as the messaging interventions increased in intensity, the rate of women attending clinic specifically to receive a pOBU increased from a minimum of 0.1 (95% CI 0.0 to 2.9) in the control to a maximum of 46.0 (95% CI 31.5 to 67.0) in intervention C.

We conducted an exit survey that gave the opportunity for clients to make comments about their experience. Although visualization of the unborn child is considered to be a curse in some cultures, there was no evidence of this belief in this region. In total, 84/149 (56.38%) respondents provided a voluntary free-text description of their experience with pOBU in the exit survey (S2 File). Of all descriptors, 96/97 (99%) were positive. The most frequently used words were “happy”, “liked” and “good” (28, 23 and 14 times respectively). Only one negative word was used in one instance throughout all responses. One woman declined ultrasound because she was told by a local woman that “it was not good for the baby”. Two other women who underwent an ultrasound recounted that “people say if you go in scan, you become weak” and “people have said that if you go in scan, your years of living have been reduced”. However, both study participants ultimately came to ANC to receive a pOBU because they “wanted to see the baby”.

## Discussion

To our knowledge this is the first randomized trial exploring the relationship between advertising availability of pOBU and attendance at ANC. We demonstrated that advertising pOBU by radio messaging significantly increased ANC attendance. In particular, our approach of incentivizing care with the message that pOBU allows women to “see their baby” resulted in significantly increasing ANC attendance in the most vulnerable population of women—those rural poor who had previously seen a TBA. We believe that this fits in line with the literature and expert opinion regarding rural women feeling isolated from high quality healthcare, being mistrustful of modern medicine, and highlights the utility of pOBU in motivating these women to attend care.[9–12,24]

It has previously been shown that pOBU is a cost effective and readily utilizable resource in low-income settings that can help to reduce maternal anxiety.[21,22,28,29] In our study, a single charged battery pack was sufficient to power one pOBU for an entire clinic day, with the ability to scan over 40 women. Utilizing pOBU does require initial investment costs, and technical and logistic factors such as training ultrasonographers and obtaining vehicles necessary to access isolated communities.

A major difficulty in our study was that the message of ultrasound availability was not being adequately delivered to the pregnant women. This became apparent in the first community which had pOBU advertised by WOM (intervention A). Despite WOM advertising of pOBU, very few women who attended ANC in intervention A were aware of pOBU availability, or meaning. As a result, that evening a decision was made to immediately broadcast a radio message explaining that an ANC would be conducted the following day, with no mention of pOBU by radio and continued messaging of pOBU by WOM (intervention B). The clinic repeated the following day, with similar attendance rates and no significant increase in study participants attending clinic for pOBU (Table 3). The following evening another radio message was made, this time advertising an ANC, as well as a brief message regarding pOBU and the ability to “see a picture of your baby” (intervention C).

This latter radio message, which explained the meaning of ultrasound, created an increase by the following morning in the number of women attending ANC because they wanted to have pOBU when compared with controls, and when compared with both other intervention arms (Table 3). As only one community received intervention A and one received intervention B, our study was underpowered to detect differences in outcomes between the various intervention arms. Future studies should budget resources and time such that they are able to hold different intervention arms on multiple days in multiple communities, this would allow for increased power to detect differences between intervention arms, and time to assess the durability of radio messaging.

Our study has some additional limitations. It was conducted across homogenous sub-counties that are rural and isolated. This was useful to ensure that blinding of pOBU was successful and for comparison amongst arms, but should be interpreted with caution in applying our results to communities outside of the Kabale District. Although isolated, these communities are still ethnically and culturally similar to the approximately 1.2 million inhabitants of the Kigezi Region, as well as neighbouring regions of Rwanda, and the Democratic Republic of Congo.[30] Additionally, as there had been no census performed in Uganda since 1994 we did not have data on the actual number of women who were aware that they were pregnant in each community. We therefore substituted this with the total number of women who registered for a first ANC visit in 2013/14 in those communities in order to estimate the number who were actually aware that they were pregnant. Two methods of sensitivity analysis were conducted, one using the number of women who delivered babies at a government facility in these communities, and another using an average of two years of data (2012/13 and 2013/14) for women who registered for a first ANC visit. This analysis showed the same findings as the initial denominator, and therefore we feel that these findings are robust (S1 Table). A pre-post design in the intervention arms would have been helpful to further strengthen the analysis. Finally, although we measured a large increase in clinic attendance based on advertisement of pOBU by radio, we cannot confirm that this effect would continue and encourage women to reach four recommended visits during prenatal care. Additionally, it is unclear if the effect would be replicated in subsequent pregnancies, or would in fact lead to delivery in a HCF. However, it is notable that a previous quasi-experimental study in Uganda suggested that introduction of obsteric ultrasound in a clinic was associated with subsequent increased numbers of women attending second, third and fourth antenatal care visits and increased numbers of deliveries at the clinic.[31] As well, there is currently a protocol in place for a cluster-randomized trial set out to examine the relationship of ANC attendance and delivery at HCF.[32]

## Conclusions

We have demonstrated that portable obstetric ultrasound can serve as an important tool to rapidly increase uptake of ANC services in rural disadvantaged women when combined with radio messaging. Our findings have the potential to inform public health agencies, and improve ANC attendance in resource poor settings. Given that pOBU is not common in Uganda, we hope that this study will serve as impetus for ministries of health and existing NGOs to begin actively utilizing pOBU in their ANC clinics. Subsequently, we hope that more comprehensive studies will be conducted to assess the efficacy of pOBU in not only increasing uptake of ANC services, but in bringing women into the healthcare system and encouraging them to deliver at HCFs.

## Supporting information

S1 Fig Map of Kabale region, broken down by sub-counties. (PDF)

S2 Fig Participant flow through clinic. (DOCX)

S1 File Copy of entrance survey questions. (DOCX)

S2 File Description of qualitative exit survey findings. (PDF)

S3 File CONSORT 2010 checklist. (DOC)

S4 File MUST IRC ethical applications form. (DOCX)

S1 Table Sensitivty analysis of data using combined 2013–14 health facility attendance. (DOCX)
