# Supplementary figures and images for: Effectiveness of advertising availability of prenatal ultrasound on uptake of antenatal care in rural Uganda: A cluster randomized trial
Source: PLoS One. 2017 Apr 12;12(4):e0175440. doi: 10.1371/journal.pone.0175440 (PMC5389838; doi:10.1371/journal.pone.0175440)

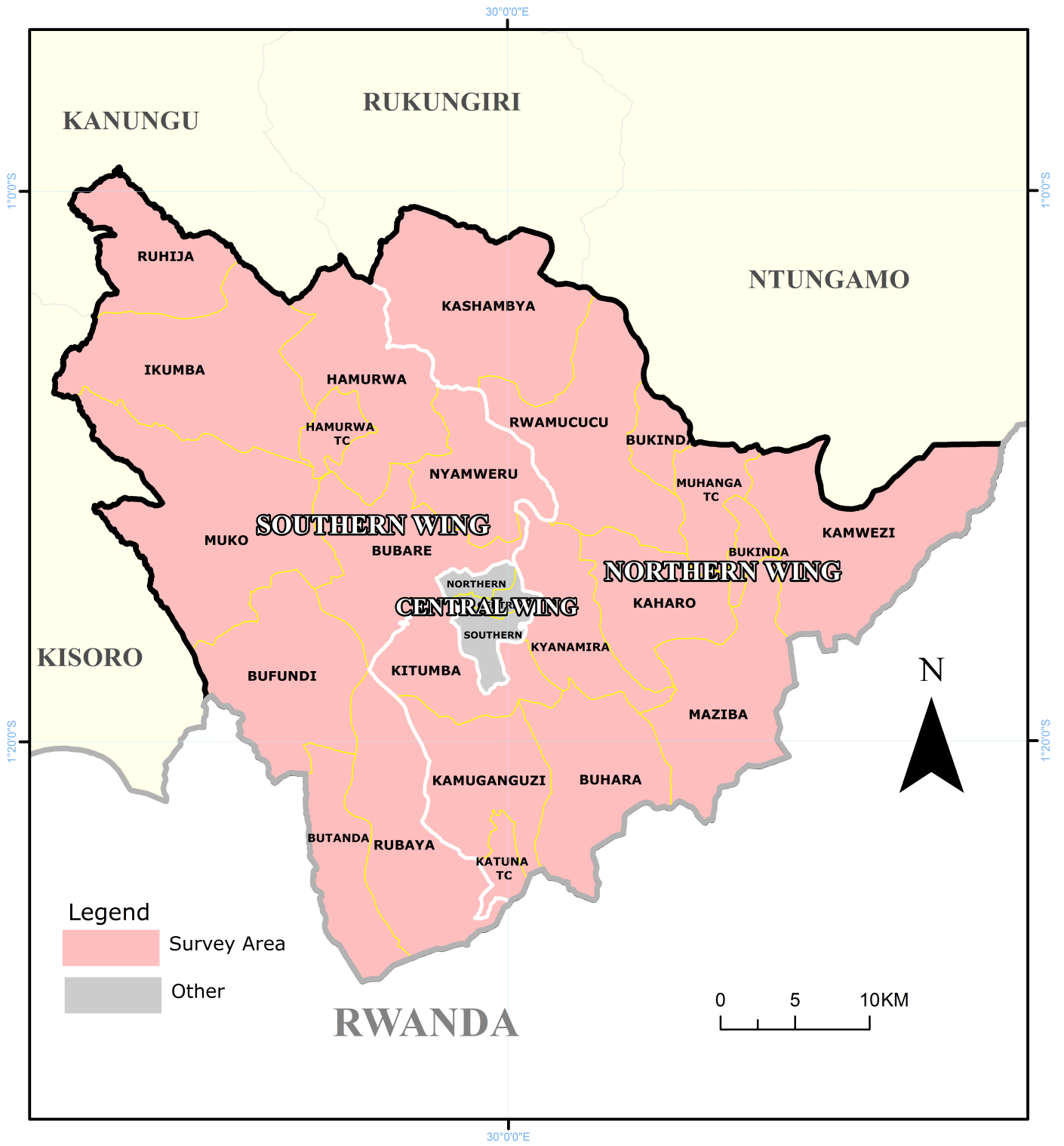

Supplement: S1 Fig — (PDF) [file pone.0175440.s001.pdf]
