# Supplementary material for: Effectiveness of advertising availability of prenatal ultrasound on uptake of antenatal care in rural Uganda: A cluster randomized trial
Source: PLoS One. 2017 Apr 12;12(4):e0175440. doi: 10.1371/journal.pone.0175440 (PMC5389838; doi:10.1371/journal.pone.0175440)
Supplement: S2 Fig — (DOCX) [file pone.0175440.s002.docx]

**Patient presents to registration**

**Pre-test counselling for HIV followed by bloodletting**

**Debriefing form completed**

**Consent form completed**

**Entrance survey completed**

**Goes for free portable obstetric ultrasound scan**

**Results are positive for HIV**

**Results are positive for Hepatitis B**

**Results are positive for malaria or syphilis**

**Ultrasound scan is concerning**

**Appropriate counselling, treatment and/or referral made**

**Receives family planning**

**iPT for malaria, folic acid/iron supplementation +/- personalized medical and/or dental care provided**

**Exit Survey completed**

**Discharge Home**

**Figure 1. Flow chart depicting patient experience in structured maternal health clinic (sMHC)**

**Appendix B:** Structure of data collection and patient flow through the antenatal care clinic.
