# Supplementary material for: Effectiveness of advertising availability of prenatal ultrasound on uptake of antenatal care in rural Uganda: A cluster randomized trial
Source: PLoS One. 2017 Apr 12;12(4):e0175440. doi: 10.1371/journal.pone.0175440 (PMC5389838; doi:10.1371/journal.pone.0175440)
Supplement: S1 File — (DOCX) [file pone.0175440.s003.docx]

**Maternal Entrance Survey Questions:**

1. Name
2. Health Unit
3. Village
4. Religion
5. Occupation

1= peasant, 2= housewife, 3=teacher, 4=self employed, 5=saloon worker, 6=professional degree, 7=farmer

1. Relationship

1=married, 2=single, 3=stable heterosexual relationship

1. Number of previous boyfriends and/or marriages
2. Next of Kin
3. Highest level of Education

99 = no school, 1=did not complete primary school, 10 = high school 11= completed high school 12=started post secondary 13 = completed post secondary 14=primary school only

1. Did you come because of the ultrasound

1=yes, 2=no

1. Why did you come to this clinic today?
2. Number of pregnancies

99 = no answer

1. Number of deliveries
2. Number of live births
3. Number of miscarriages
4. Number of induced abortions
5. Number of vaginal births
6. Number of c-setions
7. Number of deliveries at home
8. Number of deliveries with traditional healer
9. Complications of pregnancy or delivery at home

1=yes 2=no 99 = don't know

1. If yes, did you go to a hospital for treatment?

1= yes, 2= no, 99 = don't know

1. If yes to complication, was there:

fever (=1), cough (=2), heavy excessive bleeding (=3), vaginal tear (=4)

1. Number of deliveries at hospital
2. Family planning

1=yes, 2=no, 99=don't know

1. Condoms = 1, OCP = 2, injections = 3, IUD= 4, rhythm method = 5 don't know = 99
2. Screen for cervical cancer

1=yes, 2=no, 99= don't know

1. Did you put anything onto the umbilical cord after delivery

1=yes, 2=no

1. Comments for AC
   1. 1=onion leaves, 2=spit/saliva, 3=vaseline/petroleum jelly, 4=dirt/dust/ash, 5=baby powder, 6=normal saline, 7=traditional herbs, 8=ghee (clarified butter)
2. Obs U/S before
   1. 1=yes, 2=no
3. HIV

1=yes, 2=no

1. If yes to HIV on ARV
   1. 1=yes, 2=no
2. TB

1=yes, 2=no

1. If yes to TB received treatment?

1=yes, 2=no

1. Seizures

1=yes, 2=no

1. If yes to seizures on treatment

1=yes, 2=no

1. Sickle cell

1=yes, 2=no

1. If yes to sickle cell, any crisis during pregnancy?

1=yes, 2=no

1. Syphilis

1=yes, 2=no

1. If yes to syphilis, was treated/is on treatment?

1=yes, 2=no

1. DM

1=yes, 2=no

1. If yes to DM, was on treatment/is on treatment?

1=yes, 2=no

1. Hypertension

1=yes, 2=no

1. If yes to HTN is on treatment?

1=yes, 2=no

1. Malaria during pregnancy?

1=yes, 2=no

1. Fansidar?

1=yes, 2=no

1. Twins

1=yes, 2=no

1. Have you used herbs for vaginal cleansing?

1=yes, 2=no

1. Hepatitis B

1=yes, 2=no

1. Have you seen a traditional healer?

1=yes, 2=no

1. First day of LMP
2. EDC
3. Weeks gestation
4. Have you seen a dr, midwife or nurse during the pregnancy?

1=yes, 2=no

1. If yes to seeing a healthcare worker during pregnancy, how many times?
2. Contractions?

1=yes, 2=no

1. Hospitalized for bleeding

1=yes, 2=no

1. Fever

1=yes, 2=no

1. Cough

1=yes, 2=no

1. Water broken

1=yes, 2=no

1. Did you know there would be free ob ultrasound

1=yes, 2=no

**Maternal Exit Survey:**

1. Name
2. Midwife exam
3. There are currently many healthcare centers in and around the Kabale Region, do you plan on delivering your baby at a healthcare center or at home? (1= healthcenter, 2=home)
4. If at home why?
5. If at healthcare center why?
6. Have you changed your answer compared to before you came to clinic? (1=yes, 2=no)
7. If yes why?
8. US result
   1. 1=normal 2=Abnormal
9. If abnormality, what was it?
10. US estimated gestational age ( in weeks)
11. HIV
    1. 1=negative, 2=positive
12. Hbg
13. Syhphilis
    1. 1=negative, 2=positive
14. Malaria
    1. Negative, 2=positive
15. Hepatitis B
    1. negative=1, positive =2
16. Glucose
17. What do you think about the US?
