# Supplementary material for: Effectiveness of advertising availability of prenatal ultrasound on uptake of antenatal care in rural Uganda: A cluster randomized trial
Source: PLoS One. 2017 Apr 12;12(4):e0175440. doi: 10.1371/journal.pone.0175440 (PMC5389838; doi:10.1371/journal.pone.0175440)
Supplement: S2 File — (PDF) [file pone.0175440.s004.pdf]

Descriptor[1]	Frequency	Proportion of Positive Descriptors	Proportion of Negative Descriptors	Proportion of Total Descriptors
**Happy**	28	29.2%		28.9%
**Liked**	23	24.0%		23.7%
**Good**	14	14.6%		14.4%
**Okay**	4	4.2%		4.1%
**Well**	4	4.2%		4.1%
**Like**	2	2.1%		2.1%
**Normal**	3	3.1%		3.1%
**Enjoyed**	3	3.1%		3.1%
**Relieved**	2	2.1%		2.1%
**Useful**	2	2.1%	N/A	2.1%
**Likes**	2	2.1%		2.1%
**Eager**	1	1.0%		1.0%
**Comfortably**	1	1.0%		1.0%
**Appreciated**	1	1.0%		1.0%
**Excellent**	1	1.0%		1.0%
**Reassured**	1	1.0%		1.0%
**Safe**	1	1.0%		1.0%
**Nice**	1	1.0%		1.0%
**Welcomed**	1	1.0%		1.0%
**Grateful**	1	1.0%		1.0%
**Panicking**	1	N/A	100.0%	1.0%
