# Supplementary material for: Effectiveness of advertising availability of prenatal ultrasound on uptake of antenatal care in rural Uganda: A cluster randomized trial
Source: PLoS One. 2017 Apr 12;12(4):e0175440. doi: 10.1371/journal.pone.0175440 (PMC5389838; doi:10.1371/journal.pone.0175440)
Supplement: S4 File — (DOCX) [file pone.0175440.s006.docx]

| **FOR OFFICE USE ONLY** | Date of submission | Date considered | Approval granted? |
| --- | --- | --- | --- |
| Application No. ***(Yr/No)*** |  |  | yes / no |
| Signature:  (MUST-IRC Chair) |  |  |  |

THIS FORM MUST BE TYPEWRITTEN

# MBARARA UNIVERSITY OF SCIENCE AND TECHNOLOGY

**APPLICATION FORM FOR INSTITUTIONAL ETHICAL APPROVAL**

**ALL** QUESTIONS MUST BE ANSWERED. ANY FORM STATING "SEE PROTOCOL" WILL BE RETURNED. (This form must stand complete in itself).

PLEASE PROVIDE COPIES OF THIS FORM AND THE ORIGINAL PROPOSAL AS STATED IN THE GUIDELINE

AS FAR AS POSSIBLE YOU SHOULD RESTRICT ALL ENTRIES TO THE SPACE PROVIDED ON THIS FORM

Please use a typing font that is easily distinguishable from the questions of the form

NB This form is available on diskette from the MUST-IRC Office

| **NAME OF APPLICANT: Geoffrey Anguyo** |
| --- |

Have you submitted this proposal to the relevant Faculty/Institute Research Committee before?

No Yes Faculty:

1

No

Date and outcome:

If you are re-submitting a proposal, please emphasize how the proposal has been amended in the light of previous recommendations from the Faculty Research Committee or Institutional Ethical Review Committee.

|  |
| --- |

If this proposal is for work that will go towards a higher degree (e.g. M.Med or PhD), please state name and Department of Supervisor(s):

**SECTION A**

**STUDY OUTLINE**

**A.1 TITLE OF PROJECT:** Utilization of portable ultrasound as a means to increase uptake of antenatal care services in rural Uganda

**A.2 SUMMARY**

Explain why this study is being conducted, using lay terminology.

*Guidance note:*

*Please convey what you think is the importance of the research and WHY it is being carried out.*

The national average for the percentage of women who go to at least on antenatal care (ANC) clinic in Uganda is close to 95%, but in the Kabale region we think it is closer to only 60%. This is thought be a result of isolated mountainous topography of villages combined with a deep mistrust of the governmental healthcare system compounded by a high reliance on traditional healers. It is well known that screening of women who are pregnant can help to detect problems that could affect the mother’s health as well as be a risk to the unborn baby. Many of these problems are treatable and some are curable, which could help the mothers and infants health. We plan to entice pregnant women on testing for HIV, syphilis, Hepatitis B, anemia (low blood count) by offering a free portable obstetric ultrasound (fOBU). If the women are found to have any of these conditions they will be provided with the appropriate management free of charge as per Ugandan Ministry of Health Guidelines.

**A.3 OBJECTIVES**

List the major objectives/hypothesis, which have governed your choice of study design

Major Objectives:

A) To improve maternal attendance at antenatal clinics and thus achieve the following outcomes by implementing routine National Ugandan screening recommendations:

i) To reduce Mother to Child Transmission of HIV

ii) Reduce Mother to child transmission of syphilis, Hepatitis B and prevent malarial complications during pregnancy

iii) Improve maternal preference for delivery with medical professionals

B) To identify antepartum risks for increased perinatal complications by offering free ultrasound. In particular, multiple birth, breech presentation, or low-lying placenta; with arrangements to be made for delivery in specialized facilities for women who are thus found to be in need.

Hypothesis of the study:

Although antenatal care in rural Uganda is hindered by geographic constraints, lack of personal finances and government health care infrastructure, cultural attitudes towards western medicine are also an important concern. Many women do not present for antenatal care as they feel that “just blood testing and tablets” are not reason enough to present for antenatal care. Offering free obstetric ultrasound will significantly increase the number of women in rural Uganda who present for antenatal care.

**A.4 METHODOLOGY**

Outline how you intend to achieve the objectives of the study.

*Guidance notes:*

*For each objective/hypothesis:-*

- *define the target population*
- *describe how the sample(s) is(are) to be recruited from the target population(s)*

*Even if the main thrust of the research is biomedical, the rationale behind your use of social science methods (e.g. patient interviews) should be clear.*

We intend to achieve the objectives of our study through two different research aspects, one focusing on non-clustered randomization (sample size of 288) and other looking specifically at randomized clusters (sample size of 550). We have clustered at the community level to observe for an effect of fOBU on increasing overall ANC attendance. We have randomized at the patient level for assessment of the efficacy of medical/dental outreach with fOBU to change patient preference for delivering in a health care center.

Communities in the Kabale District will be assessed for population size, demographics and birth rate. Communities that have similar characteristics will be selected and contacted to engage in the study. Communities who agree to participate in this study will be contacted and community leaders will make announcements at local schools, churches, and community events regarding the upcoming free antenatal clinics. During these announcements the community will be told either a) that there will be structured maternal health camps (sMHC) offering free obstetric ultrasound (OBU) or b) that there will be sMHCs with no mention of the fOBU. In actuality, to assure that all women are given the benefit of optimal care, all SMHCs will provide fOBU, but only the advertising messages will differ between groups. Communities will be stratified by catchment population (small, medium or large) prior to randomization and assignment to the intervention or control group. Additionally, we will be collecting demographic data on registration to clinic and asking a pre clinic questionnaire to assess maternal feelings towards the healthcare system, and their preferences for where to deliver their baby (at a healthcare center or at home).

**A.5 PARTICIPANTS**

Please provide the following information on the participants with/from whom you expect to be collecting data:

A.5.1 Age / Sex: (please enter the expected number in each of the boxes)

|  | Neonates  (<28 days) | Infants  (1-11 months) | Young children  (1-9 years) | Adolescents  (10-17 years) | Adults  (18 yrs & above) |
| --- | --- | --- | --- | --- | --- |
| **Males** | **0** | **0** | **0** | **0** | **0** |
| **Females** | **0** | **0** | **0** | **0** | **550** |

A.5.2 What specific measures are in place to take into account women of childbearing age?

*Guidance notes:*

*Pregnant women may have different responses to disease processes*

*The developing foetus may be particularly vulnerable in intervention trials*

The main goals and objectives of this study revolve around recruiting pregnant women. However, given that all of the interventions being offered are standard of care and not experimental or dangerous in any way there will be minimal risk to this category of patients.

A.5.3 Describe how and where the participants are to be recruited?

*Guidance notes:*

*This is distinct from the statistical sampling method described in A.4. You should outline the procedures for recruitment of each group of participants, include details on:*

- *the setting (e.g. Country, Town, District, on the ward, out-patient department ,in the home)*
- *inclusion and exclusion criteria for selection, if relevant (e.g. “Women of child-bearing age will be excluded”)*

As described above, participants will be recruited from communities in the Kabale Region by announcements that will be made by community leaders at local schools, churches, and community events regarding the upcoming free antenatal clinics.

Inclusion criteria include:

The participants to must all be residents of the Kabale Region. All pregnant women who present to one of the sMHCs will be included.

Exclusion criteria include:

Any males or non-pregnant females who attend the health camps will be excluded from the study.*

Notes:

*All participants excluded from the study will still offered free medical and dental care as part of a mobile free medical/dental clinic that will be offered in the same community at the same time but in a neighbouring location.

A.5.4 Please justify your choice of sample size (as described in A.4)

Sample size calculations were considered for two of the primary outcomes. For the first outcome – looking to determine the efficacy of portable ultrasound in improving maternal uptake of antenatal care due to portable ultrasound being offered – a simple randomization based on communities will be utilized. For the additional outcome of eliciting a change in patient preference for delivering at a healthcare facility before and after a rural outreach clinic the communities will be both clustered and randomized. In this case a design effect is added to account for the clustering for this patient-level outcome.

1. Facility based uptake of ANC (no clustering): At a two-sided alpha of 0.05 and holding power at 80%, with p1 of .70 and p2 of 0.52 [1], then 144 pregnant women will be required in each of the control and intervention groups. Inflating this by 25% to prepare for any non-consent and/or non-response, the required sample increases to 180 women per group. From experience gained in our pilot study, we anticipate that health facilities will enroll a greater number of women, therefore our sample will provide more than adequate power. We will conduct power calculations during analysis, however, we anticipate power of greater than 90%.
2. Patient-level outcomes (clustered): At a two-sided alpha of 0.05 and holding power at 80%, using an estimated intracluster coefficient (ICC) of 0.1 [2], with p1 of 0.95 and p2 of 0.85, and an average cluster size of 60 pregnant women (m), then 225 pregnant women will be required per study arm, which is equivalent to needing 4 clusters per group. Inflating this by 25% to account for potential non-response gives 282 pregnant women per group.

Notes:

1. Rate of uptake of antenatal care (at least 1 ANC visit) in pilot studies in our community
2. Generally, ICCs are not known prior to study start. For ICC assumption, it is generally accepted that clinical outcomes have ICCs of around .05; whereas process outcomes (which our research is studying) range from 0.05 to 0.15; we have used 0.1 in our calculations.

Reference: <http://www.who.int/reproductivehealth/topics/best_practices/intracluster_correlation.pdf>

**A.6 PROCEDURES**

A.6.1What procedures or methods will be employed in the collection of data (e.g. patient interviews / focus group discussions / blood sampling / biopsies) and by whom (e.g. experienced facilitator / social scientist / teacher/ qualified doctor / nurse, auxiliary, etc.)?

Attach additional sheets if necessary.

| **Procedure** | **To be carried out by:** |
| --- | --- |
| **Maternal survey** | **Qualified research associate** |
| **Exit Survey** | **Qualified research associate** |
|  |  |
|  |  |
|  |  |
|  |  |
|  |  |

A.6.2 State the extent to which the procedures to be used are a part of usual clinical management (if appropriate).

In this case the intervention – a free obstetric ultrasound – has low risk for potential adverse outcomes. False positive screening ultrasound identifying non-existent congenital anomalies may lead to maternal concern. Any women who have an examination that demonstrates concerning findings will be transferred free of charge to a tertiary care facility for obstetrical consultation and confirmatory testing. Thus, the benefit of early identification and management of true positive tests with prevention of maternal and fetal adverse outcomes will outweigh the potential risk of rare false positive testing.

A.6.3 Please indicate that the persons identified in A.6.1 are competent to carry out these procedures. List any training of staff that may be required prior to commencement of the study.

Albert Co: Bachelor of Sciences, major in Biology, with a minor in neurosciences

Ankur Gill: Bachelor of Sciences, major in Biology

No training will be required prior to the commencement of the study

**A.7 ANALYSIS**

A.7.1 What are the major statistical (or other) methods that you intend to use to analyse the data to

fulfill each of the objectives/hypothesis stated in A.3

As it is recommended in cRCT, and in particular with relatively few numbers of clusters, to assess the effectiveness of randomization we will first examine the baseline characteristics of the health centre clusters and the individual patients across intervention and control groups. Analyses will be done at both health facility and individual (patient) levels, adjusted for clustering. Health center level analyses will use multivariable linear regression to compare uptake between the intervention and control arms, adjusting for potential confounders. At the patient level, to adjust for cluster randomization, we will use generalized estimating equation binomial regression models with log link and exchangeable correlation structures to estimate the relative risks (RR) and 95% confidence intervals for rates of patient preference for delivery site between the intervention group (receive fOBU messaging) with the control group as a reference. STATA version 9.2 (Stata Corp, College Station, TX) or equivalent will be used for all analyses.

**A.8 QUALITY ASSURANCE**

A.8.1 What procedures are in place to ensure the quality of the data?

*Guidance notes:*

*For qualitative data (for example) what procedures will be used to check translations or compare data obtained from different sources?*

*For quantitative data (for example) how will transcription errors be minimised?*

*Give some detail on how methods are going to be piloted, if appropriate*

The primary objective of our study is to assess the number of women who present to ANCs at the different facilities with or without the intervention. There will be ample staff available at triage to capture all of the patients who present to each clinic thus ensuring that the maximal data will be captured at each sMHC.

The same two research associates will deliver the maternal survey to every patient enrolled into the study with the help of the same two translators. This will ensure consistency in the questions being asked and the answers that are translated. The vast majority of the answers will be very simple “yes or no” and thus will be subject to very little inter-interviewer discrepancy.

**A.9 DISSEMINATION OF RESULTS**

Please outline what plans you have for dissemination of results.

*Guidance notes:*

*Where possible a mechanism should be in place to inform study participants of the outcomes of the study.*

*It is important that study findings are made known to local services / policy makers before they are discussed (e.g.) at international scientific meetings*

Data regarding the number of women screened and the efficacy of ultrasound in attracting participants will be provided in informational sessions to community leaders and then to church and school groups. If the ultrasound is found to either A) identify important obstetrical complications that require intervention in one or more patients or B) enhance maternal presentation for care then the investigators undertake to carry out a fundraising campaign in Canada to purchase portable ultrasound devices and train local staff in the use of ultrasound at the end of the project. This will enable the community to have ongoing benefit from this intervention.

**SECTION B**

**CONSEQUENCES FOR THE LOCAL COMMUNITY / ENVIRONMENT AND PARTICIPANTS**

B.1 Outline the potential adverse effects, discomfort or risks that may result from the study in the following areas:

B.1.1 Participants

*Guidance note:*

*In addition to the physical effects of tissue sampling (for example blood sampling) it should be borne in mind that interviews and focus group discussions may sometimes trigger painful or distressing memories (e.g. questions about sexual practice or the death of a child)*

There is a potential for psychological/emotional risks on the participants from feeling embarrassed and/or uncomfortable with the ultrasound procedure. As well, there is a potential risk for false positive detection of fetal abnormality/maternal complication of pregnancy. HIV testing will be conducted, there is a risk for psychological well-being from this testing.

B.1.2 Investigators

*Guidance notes:*

*Include here (for example)*

- *the biomedical risks to investigators (including local staff) involved in tissue sampling (e.g. Hepatitis B, HIV)*
- *the psychological consequences for social science investigators exposed to narratives of violence or severe grief*
- *the risks from the environment (e.g. in a war zone)*

Given the nature of this study it is not anticipated that there will be any risk to the investigators.

B.1.3 Members of the public

Given the nature of this study it is not anticipated that there will be any risk to members of the public.

**B.2 Outline what steps will be taken to minimize the adverse effects, discomfort or risks described above.**

B.2.1 For participants

*Guidance notes:*

*In biomedical research, appropriate use of anesthesia prior to procedures (for example) is important.*

*For social science research it may be necessary to ensure that counseling services are available for those who re-live traumatic experiences through (for example) an in depth interview.*

1. To eliminate any emotional risks of feeling uncomfortable or embarrassed while receiving an obstetric ultrasound all scans will be conducted in a private room with a trained technician. If the technician is male gender the patient will have the right to request a female chaperone. No additional personnel will be able to observe the ultrasound scan unless they have received specific verbal consent from the patient.

Voluntary counseling and testing for HIV and sexually transmitted diseases will be provided as part of routine medical care. The risks associated with this approach are well documented but not altered by study involvement and is strongly advocated by all consensus national and international guidelines. All care will be provided as per Ugandan national guidelines.

1. In this case the intervention – a free obstetric ultrasound – has low risk for potential adverse outcomes. False positive screening ultrasound identifying non-existent congenital anomalies may lead to maternal concern. Any women who have an examination that demonstrates concerning findings will be transferred free of charge to a tertiary care facility for obstetrical consultation and confirmatory testing. Thus, the benefit of early identification and management of true positive tests with prevention of maternal and fetal adverse outcomes will outweigh the potential risk of rare false positive testing.

B.2.2 For investigators

*Guidance notes:*

*Where the research may involve adverse experiences for investigators (see B.3.2), de-briefing / support meetings may be important.*

N/a

B.2.3 For members of the public

N/a

**B.3 CONSEQUENCES FOR LOCAL HEALTH SERVICES**

B.3.1 What demands will this research place on local health services?

*Guidance notes:*

*For example, how much of a nurse’s usual work time will be taken up in acting as an interpreter for an outside investigator?*

The entirety of this study will be conducted by Kigezi Healthcare Foundation in collaboration with a Canadian not-for profit medical and dental organization TO – the WORLD (TOW). All research taking place will be conducted by staff and resources provided by TOW.

B.3.2 Detail how the design of the research project takes into account the demands described in 3.1.

*Guidance notes:*

*Disruption to routine services should be kept to a minimum.*

N/A

**B.4 CONFIDENTIALITY AND PRIVACY**

B.4.1 What steps will be taken to ensure privacy and confidentiality for participants?

Patient care data will be collected in clinical charts during the sMHC. This data will be stored in the local health care facilities as per usual practice. Specifically, all hard copy data obtained on the ground in Kabale will be stored in a locked cabinet in locked room at the KIHEFO offices, only KIHEFO medical staff have access to patient information.

Study data will be anonymized and replaced by a study number then kept on a password protected electronic database. This database will only be made available to the immediate study staff

The data will be kept for 7 years. Following this time period the protected electronic database will be deleted.

B**.5 INFORMED CONSENT**

B.5.1 Information given to participants:

Please indicate what you will tell the participants in simple language. The purpose of the study, type of questions that will be asked, and procedure or treatment which will be applied should be described and reference should be made to possible side effects, discomfort, complications and/or benefits. Please attach consent form typed on MUST-IRC official consent form.

| **It must be made clear to the participant that he/she is free to decline to participate or to withdraw at any time without suffering any disadvantage or prejudice.** |
| --- |

In the attached consent form you will find that we have explained to the patients that they are agreeing to participate in a research study regarding antenatal care in rural Uganda. We explain that they will still be able to receive all medical and dental services free of charge even if they decide to not participate in the research study. Following the completion of the consent form the patients will be asked a number of questions in our maternal survey form. Following completion of the maternal survey form the patients will be provided with a “debriefing” and final consent form. This form will explain to the patients that they were not initially informed as to the presence of ultrasound in the clinic so as to determine accurately their reason for coming to clinic that day. This form will also very clearly state that the patient has the right to refuse the ultrasound, withdraw from the study at any point, and still receive all medical and dental care free of charge at clinic that day.

B.5.2 Outline who will deliver the above information and how?

The information attached in the consent form will be delivered by highly trained and nationally certified KIHEFO nursing staff verbally and with the aid of a printed consent form.

B.5.3 Please indicate how consent will be obtained, given local circumstances.

*Guidance notes:*

*In some societies, the concept of giving consent on an individual basis is unfamiliar. It may be necessary to obtain consent both at community and individual level.*

*Obtaining consent from minors requires both consent from the guardian and, where possible, the minor.*

N/A

B.5.4 Are any inducements to be offered to either participants or the individuals who will be recruiting them? (e.g. improved patient care / cash) (please tick appropriate box)

Yes No

2

X

3

B.5.5 If yes, please give details:

N/A

B.5.6 Outline any hidden constraints to consent.

*Guidance notes:*

*Examples where hidden constraints may be important include:*

- *situations where participants are employees of the investigator*
- *patients who may feel their care could be compromised if they do not consent to research initiated by their carers.*

N/A

**SECTION C**

**RESPONSIBILITY**

C.1 Litigation:

In respect of any litigation which may result from this research

a) Who will provide compensation?

The medical doctors from Canada volunteering for the health camps are all covered for malpractice insurance by the Canadian Medical Protection Association (CMPA). The Canadian not for profit working with KIHEFO in the health camps is covered with Director and Officer Insurance in case of any litigation.

(Please provide documentary evidence where appropriate.)

b) What insurance arrangements have been made by the applicant and his/her delegated assistants?

See above

(Please ensure that any professional indemnity insurance is logged with the Director's office)

C.2 **DECLARATION: TO BE SIGNED BY MAIN APPLICANT**

- I confirm that the details of this proposal are a true representation of the research to be undertaken.

- I will ensure that the research does not deviate from the protocol described.

- If significant protocol amendments are required as the research progresses, I will submit these to the Mbarara University Faculty Medicine Research Ethics Committee for approval.

- Where an appropriate mechanism exists, I undertake to seek additional local Ethical Approval in the country(ies) where the research is to be carried out.

I expect the project to commence on (Date): February 16, 2014 and be completed by

(Date): February 23, 2014

________________________________________________________________________

Signed Date

**SECTION D**

**APPROVALS**

D.1 List research team and all collaborators.

(Please include all overseas collaborators and give their affiliations, qualifications and role in the study).

1. Dr. Michael Silverman – Board Member, TO – the WORLD, MD, FRCP, FACS, Infectious Disease Specialist, Investigator
2. Dr. William Cherniak – Executive Director, TO – the WORLD, BSc, MD, Family Medicine Resident, Investigator
3. Dr. Romina Pace – Volunteer, TO – the WORLD, MD, Internal Medicine Resident, Research Associate
4. Dr. Sumeet Sodhi – MD, MPH, CCFP, Investigator
5. Dr. Geoffrey Anguyo – Executive Director, KIHEFO, MD

**Title:** Effectiveness of advertising availability of prenatal ultrasound on uptake of antenatal care in rural Uganda: a cluster randomized trial

**1. Background**

According to statistics provided by the World Health Organization, Uganda currently has the 20^th^ highest rate of maternal mortality, and the 15^th^ highest rate of infant mortality in the world.^1^ Amongst nations in Africa, Uganda has one of the highest population growth rates, with an annual increase in 3.2% and a fertility rate of 6.7%.^1^ With approximately 1.5 million pregnancies/year, it is estimated that nearly 6000 women will die due to pregnancy related complications (16 mothers/day) – numbers which are exacerbated by poverty, malnutrition, illness, and lack of access to antenatal, peri-natal and postnatal health care services.

While maternal and child mortality is prevalent nationwide, the situation is particularly problematic in remote rural regions such as Kabale, which is located in southwestern Uganda. In contrast to the urban regions of Uganda where it is reported that close to 95% of women attend at least one antenatal care clinic (ANC)^2^ prior to delivery , current data points to only 66% presenting for ANC in rural Kabale^3^. Additionally, compared to nearly 60% of women delivering in health centers in urban areas, only 30% of births in Kabale take place in health centers, with most births occurring at home.^3,4^

Extreme poverty and malnutrition amongst women in the region have led to high rates of anemia – studies indicate that 64% of pregnant or new mothers are suffering from anemia in Kabale – in addition to high rates of HIV and malaria. As a result, 70% of the population who deliver at home in the Kabale region, are attended only by unqualified midwives who lack the necessary training and equipment to respond to high-risk delivery complications, are facing the risk of experiencing severe bleeding (caused by post-partum hemorrhage), infection (mostly after delivery), hypertensive disorders (eclampsia), obstructed labor, and ultimately, death.^4^

There are multiple contributing factors that are preventing pregnant women living in the Kabale region from receiving maternal healthcare services from trained professionals. These include cost, access, infrastructure, and cultural attitudes.

One of the greatest barriers to maternal health is geography and distance from regional centers where maternal health services are provided. The population in Kabale is situated in smaller, rural communities scattered across a geographically mountainous region. As the majority of rural communities are lacking healthcare centers and trained professionals, pregnant women can only receive trained maternal healthcare services outside their communities. However, the distance from their local communities to the regional center where the government hospital and Kigezi Healthcare Foundation’s (KIHEFO) medical and maternity center are currently operating is immense. As a result, the majority of subsistence agriculture households cannot afford the cost to travel to regional centers for antenatal, natal and postnatal care.

Cultural attitudes surrounding childbirth, mainly the fear and stigma of receiving maternal healthcare outside home communities, is also problematic to providing pregnant women with professional medical healthcare services, and overall, reducing maternal mortality. This cultural fear is generated largely by a lack of education amongst rural populations in understanding the additional risks associated with childbirth due to realities of poverty, malnutrition, disease and illness. As a result, many pregnant women who are living in rural communities maintain the preference to give birth at home, despite lacking access to trained birth attendants who can respond to high-risk complications that arise, and facing high rates of maternal mortality.

Consultations with local health care workers and community leaders during a medical mission in March 2013 suggested that a significant additional barrier to care is that local women do not appreciate the benefits of routine antepartum screening and treatment. In fact, when antenatal medical services are offered free of charge in a local context women often fail to turn out for the program. At present, local antenatal care includes a general examination including blood pressure testing, maternal hemoglobin testing, maternal voluntary HIV counseling and testing, serology for syphilis, Hepatitis B surface antigen testing, syndromic management for sexually transmitted infections and provision of free maternal iron and folate as well as presumptive treatment for malaria using fansidar as per WHO guidelines. If HIV is diagnosed, free antiretrovirals are offered as part of the national program. Similarly, benzathine penicillin G therapy is administered to patients with a positive treponemal test for syphilis with the resultant prevention of long-term maternal complications as well as the severe complications to the infant of congenital syphilis. Hepatitis B vaccine is also offered to all infants of mothers who are Hep BsAg positive (as per routine Ugandan national guidelines).

Although this comprehensive approach would offer many potential benefits to both mother and infant, local thought leaders believe that women do not see these interventions as significant, and therefore do not present for care even when mobile free clinics are brought into their communities. The impression of the local medical staff is that the importance of these “low tech” interventions are not appreciated in the general community. On the other hand these same thought leaders have suggested that women would present for care to have the concrete outcome of “seeing a picture of the baby”. In addition to being appealing to women who are pregnant for personal reasons, studies have shown that obstetric ultrasound imaging can prevent many serious complications of pregnancy (listed previously) by providing early diagnosis and intervention. For example, by providing useful information such as whether or not the mother is carrying twins, has an ectopic pregnancy or placenta previa, a mother and her partner can make an informed decision about whether or not to deliver at home with untrained professionals, or a health center where they can receive life-saving treatment.^1^ Furthermore, the World Health Organization (2003) recognizes ultrasound technology as ideally suited to low and middle income countries, as it is relatively low-cost, low input, and easily maintained and transported. Additionally, studies conducted on the use of ultrasound technology in two rural hospitals in Rwanda have indicated that after an initial training period, an ultrasound program led by local health care providers can be sustainable and lead to accurate diagnoses.^2^ Ultrasound imaging is beneficial to rural populations as it is a simple a non-invasive procedure. This helps to reduce levels of fear from women who have previously maintained their cultural preferences for receiving treatment and giving birth with untrained birth attendants in their local villages.^3^

We carried out a small pilot program in March 2013 that screened 48 antenatal women with free mobile ultrasound. We found the technology to work well within the local context and women routinely expressed their appreciation and impression that they were glad they had come and would recommend it to their peers. All of the above routine antenatal screening and interventions were offered at the same time as per routine local guidelines.

**2. Hypothesis / Objectives of the study**

Hypothesis:

Although antenatal care in rural Uganda is hindered by geographic constraints, lack of personal finances and government health care infrastructure, cultural attitudes towards western medicine are also an important concern. Many women do not present for antenatal care as they feel that “just blood testing and tablets” are not reason enough to present for antenatal care. Offering free obstetric ultrasound will significantly increase the number of women in rural Uganda who present for antenatal care.

Objectives:

A) To improve maternal attendance at antenatal clinics and thus achieve the following outcomes by implementing routine National Ugandan screening recommendations:

i) To reduce Mother to Child Transmission of HIV

ii) Reduce Mother to child transmission of syphilis, Hepatitis B and prevent malarial complications during pregnancy

iii) Improve maternal preference for delivery with medical professionals

B) Identify antepartum risks for increased perinatal complications by offering free ultrasound. In particular, multiple birth, breech presentation, or low-lying placenta; with arrangements to be made for delivery in specialized facilities for women who are thus found to be in need.

**3. Design and Methodology**

Communities in the Kabale District will be assessed for population size, demographics and birth rate. Communities that have similar characteristics will be selected and contacted to engage in the study. Communities who agree to participate in this study will be contacted and community leaders will make announcements at local schools, churches, and community events regarding the upcoming free antenatal clinics. During these announcements the community will be told either a) that there will be structured maternal health camps [sMHC] offering free obstetric ultrasound [fOBU] or b) that there will be sMHCs with no mention of the fOBU. In actuality, to assure that all women are given the benefit of optimal care, all SMHCs will provide fOBU, but only the advertising messages will differ between groups. Communities will be stratified by catchment population (small, medium or large) prior to randomization and assignment to the intervention or control group. The sMHCs will be organized in collaboration with TO – the WORLD and KIHEFO. The four-pronged approach of MTCT elimination will be followed in the design of the sMHC. This will include primary prevention of HIV, prevention of unintended pregnancies, prevention of vertical transmission and assuring linkages to care treatment and follow-up. Each sMHC will take place over a 12-hour period. All women who appear for clinic will be registered, with demographics recorded, and go through a triage process. They will then receive pre-test counseling for HIV, followed by blood sample collection for onsite testing for HIV/syphilis/hepatitis B, they will then go through family planning sessions, receive a fOBU, will be seen by a triage nurse and then be seen by an MD or DDS (Dentist) depending on their triage needs. All routine care including iron, folate and intermittent presumptive therapy for malaria will be offered free of charge. Women who are found on ultrasound screening to have potential obstetrical high risk pregnancies (low lying placenta, twins, potential birth defects) will be referred for local obstetrical services in Kabale (the regional referral center). Ultrasound will be performed using two Sonosite 180 portable ultrasonographic units operated by two technicians trained to levels of competency as dictated by national guidelines.

**4. Justification for Use of Deception**

Patients will not initially be informed of the study design, which seeks information on how availability of ultrasound will impact their decision to seek out antenatal care. They will receive a questionnaire that will seek to clarify their rationale for seeking care. As this is a major study outcome which could be impacted by knowing the study design, it will be necessary to withhold this information until after the study questionnaire has been completed, i.e. if patients are told that we are aiming to clarify whether the availability of obstetrical ultrasound leads to greater turnout, they may conclude that this is an outcome that the researchers hope to see, and thus to “help the researchers” they will report that ultrasound availability motivated their decision to present for care. Therefore, only after the patients complete their study questionnaires, will they then be informed of the study goals. We do not believe that the study would be possible without this initial (and temporary) non-disclosure, and further there is no potential negative adverse impact of the non-disclosure on the patients. It is important to note that screening obstetrical ultrasound has not previously been available in these communities. The addition of this service (which is routine in developed countries) is an enhancement in service. Therefore, not advertising the availability of the ultrasound in the half of those communities that are randomized to receiving the service but not the notification of it’s availability does not pose a reduction in care from that offered routinely in rural Uganda

**5. Inclusion/Exclusion Criteria**

Inclusion:

The participants must all be residents of the Kabale Region. All pregnant women aged 18 and above who present to one of the sMHCs will be included.

Exclusion:

Pregnant women under the age of consent (under 18 years); and any males or non-pregnant females who attend the health camps will be excluded from the study.*

Notes:

*All participants excluded from the study will still be offered free medical and dental care as part of a mobile free medical/dental clinic that will be offered in the same community at the same time but in a neighbouring location. For pregnant women under the age of 18, fOBU will also be offered.

**6. Age Range**

Based on a pilot study conducted in 2013 it has been determined that the average age range of participants will be between 18-40 years of age. Any patient presenting to clinic who is under 18 years of age will be excluded from the research study but still offered all medical and dental services.

**7. Statistical Design**

The study design is a cluster randomized controlled trial, whereby communities will be randomly assigned to two groups: the intervention communities will receive outreach messaging that includes information about the availability of fOBU included in medical/dental clinics and the control communities will receive outreach messaging about the availability of medical/dental clinics without mention of the availability of fOBU (although fOBU will be available in both study arms). Outcomes will be assessed at the patient-level (clustered) and the facility-level (ANC uptake).

**8. Recruitment Process**

As described above, participants will be recruited from communities in the Kabale Region by announcements that will be made by community leaders at local schools, churches, and community events regarding the upcoming free antenatal clinics. During these announcements the community will be told either a) that there will be structured maternal health camps (sMHC) offering free obstetric ultrasound (fOBU) or b) that there will be sMHCs with no mention of the fOBU. The recruitment materials will all be in the local language, and will be translated by the local health care workers who are fluent in English and the local Luchiga dialect.

**9. Data Collection**

Patient care data will be collected in clinical charts during the sMHC. This data will be stored in the local health care facilities as per usual practice. Specifically, all hard copy data obtained on the ground in Kabale will be stored in a locked cabinet in locked room at the KIHEFO offices, only KIHEFO medical staff have access to patient information.

Study data will be anonymized and replaced by a study number then kept on a password protected electronic database and analyzed using SPSS. This database will only be made available to the immediate study staff

The data will be kept for 7 years. Following this time period the protected electronic database will be deleted.

**10. Consent Process**

All patients who are seen will be voluntarily attending the sMHC, they will be consented to undergo a fOBU upon checking in at triage and again by the technician. Consents forms will be written in both English and Luchiga and they will be read to patients by KIHEFO staff for those who are not literate in either language.

**11. Risk and Benefits**

Possible Risks**:**

(i) Physical risks (e.g., any bodily contact or administration of any substance: Yes  No x

(ii) Psychological/emotional risks (e.g., feeling uncomfortable, embarrassed, or upset): Yes x No

(iii) Social risks (e.g., loss of status, privacy and/or reputation): Yes  No x

(iv) Legal risks (e.g., apprehension or arrest, subpoena): Yes  No x

Notes:

1. To eliminate any emotional risks of feeling uncomfortable or embarrassed while receiving an obstetric ultrasound all scans will be conducted in a private room with a trained technician. If the technician is male gender the patient will be offered a female chaperone. No additional personnel will be able to observe the ultrasound scan unless they have received specific verbal consent from the patient.

Voluntary counseling and testing for HIV and sexually transmitted diseases will be provided as part of routine medical care. The risks associated with this approach are well documented but not altered by study involvement and is strongly advocated by all consensus national and international guidelines. All care will be provided as per Ugandan national guidelines.

1. In this case the intervention – a free obstetric ultrasound – has low risk for potential adverse outcomes. False positive screening ultrasound identifying non-existent congenital anomalies may lead to maternal concern. Any women who have an examination that demonstrates concerning findings will be transferred free of charge to a tertiary care facility for obstetrical consultation and confirmatory testing. Thus, the benefit of early identification and management of true positive tests with prevention of maternal and fetal adverse outcomes will outweigh the potential risk of rare false positive testing.

Possible Benefits:

The patients will directly benefit from being involved in this project as they will receive free medical and dental care including an obstetric ultrasound. The community will gain a large benefit by having its members leave with improved health and psychological well being of having received medical and dental care. By engaging in this study participants will be providing data on the benefits of maternal screening utilizing portable ultrasound. It will provide the ability to prove that offering free ultrasound scans can increase turnout to maternal care clinics.

**12. Feedback to Communities**

Data regarding the number of women screened and the efficacy of ultrasound in attracting participants will be provided in informational sessions to community leaders and then to church and school groups. If the ultrasound is found to either A) identify important obstetrical complications that require intervention in one or more patients or B) enhance maternal presentation for care then the investigators undertake to carry out a fundraising campaign in Canada to purchase portable ultrasound devices and train local staff in the use of ultrasound at the end of the project. This will enable the community to have ongoing benefit from this intervention.

The study results will also be presented at an international meeting and will be submitted for publication in a peer reviewed journal.

**13. References**

1. Yaw A.W., Alexander T.O., and Edward T.D. The Role of Obstetric Ultrasound in Reducing Maternal and Perinatal Mortality, Ultrasound Imaging - Medical Applications, InTech, Accessed March, 2013. Available from: <http://www.intechopen.com/books/ultrasound-imagingmedical-applications/the-role-of-obstetric-ultrasound-in-reducing-maternal-and-perinatal-mortality>.
2. Shah S.P., Epino H., Bukhman G., Umulisa I., Dushimiyimana J.M., Reichman A., Noble V.E. Impact of the introduction of ultrasound services in a limited resource setting: rural Rwanda. BMC International Health Human Rights. 2009;27:9-4
3. Maternal Health: Investing in the Lifeline of Healthy Societies and Economies. Africa Progress Panel Position Piece. September 2010.
