# Supplementary material for: Effectiveness of advertising availability of prenatal ultrasound on uptake of antenatal care in rural Uganda: A cluster randomized trial
Source: PLoS One. 2017 Apr 12;12(4):e0175440. doi: 10.1371/journal.pone.0175440 (PMC5389838; doi:10.1371/journal.pone.0175440)
Supplement: S1 Table — (DOCX) [file pone.0175440.s007.docx]

Appendix E: Sensitivity analysis of rates and rate ratios using combined data from 2012-13 and 2013-14 for first time attendance to ANC at a government healthcare facility as denominator.

|  | **Control** | | | | **Intervention A** | **Intervention B** | **Intervention C** | |
| --- | --- | --- | --- | --- | --- | --- | --- | --- |
|  | Days 1-4  ANC=WOM | | | | Day 5  ANC=WOM pOBU=WOM | Day 6  ANC=(WOM+radio)  pOBU=WOM | Day 7-8  ANC=(WOM+radio)  pOBU=(WOM+radio) | |
| Clinic/village name | Hamurwa | Ruhija | Ikumba | Muko | Katanga | Kyanamira | Nyakigugwe | Mwisi |
| Number women attending first ANC with government (2012-2014) | 1970 | 658 | 937 | 2147 | 688 | 525 | 522 | 401 |
| Number attending | 14 | 15 | 16 | 14 | 16 | 7 | 44 | 31 |
| Rate attendance in clinic | 7.1 | 22.8 | 17.1 | 6.5 | 23.3 | 13.3 | 84.3 | 77.3 |
| Rate attendance in clinic | 10.3  (5.9, 18.1) | | | | 23.3  (8.0, 68.0) | 13.3  (2.6, 67.5) | 81.3  (49.5, 133.3) | |
| Number first ANC | 1 | 5 | 10 | 5 | 4 | 1 | 11 | 5 |
| Rate first ANC attendance in clinic | 0.5 | 7.6 | 10.7 | 2.3 | 5.8 | 1.9 | 21.1 | 12.5 |
| Rate of first ANC attendance clinic | 3.7  (1.3, 10.2) | | | | 5.8  (0.6, 59.8) | 1.9  (0.01, 201.7) | 17.3  (5.4, 55.6) | |
| Number seeing traditional healer | 2 | 4 | 0 | 2 | 1 | 2 | 7 | 8 |
| Rate seeing traditional healer | 1.0 | 6.1 | 0 | 0.9 | 1.5 | 3.8 | 13.4 | 20.0 |
| Rate seeing traditional healer | 1.4  (0.4, 4.8) | | | | 1.4  (0.04, 48.0) | 3.8  (0.3, 45.2) | 16.3  (6.6, 40.1) | |
| Number stating they came to clinic because of pOBU | 0 | 0 | 1 | 0 | 5 | 5 | 30 | 23 |
| Rate stating they came to clinic because of pOBU | 0 | 0 | 1.1 | 0 | 7.3 | 9.5 | 57.5 | 57.4 |
| Rate stating they came to clinic because of pOBU | 0.2  (0.02, 1.6) | | | | 7.3  (2.7, 19.6) | 9.5  (3.5, 25.6) | 57.4  (42.4, 77.8) | |

| **Arm of Trial** | **Primary Outcome** | **Secondary Outcomes** | | |
| --- | --- | --- | --- | --- |
|  | Rate Ratio Attending | Rate Ratio Attending First ANC | Rate Ratio Seeing Traditional Healer | Rate Ratio Stating they Came for POBU |
| A versus Control | 2.25  (0.67, 7.54)  P=0.1883 | 1.58  (0.12, 20.12)  P=0.7240 | 1.04  (0.03, 42.39)  P=0.9844 | 41.51  (3.68, 468.42)  P=0.0026 |
| B versus Control | 1.29  (0.23, 7.17)  P=0.7705 | 0.52  (0.01, 61.23)  P=0.7871 | 2.72  (0.17, 43.20)  P=0.4782 | 54.40  (4.82, 613.85)  *P=0.0012 |
| C versus Control | 7.87  (3.73, 16.60)  *P<0.0001 | 4.72  (1.01, 22.15)  P=0.0495 | 11.60  (2.51, 53.66)  *P=0.0017 | 327.99  (35.16, 3059.47)  *P=0.0001 |
| B versus A | 0.57  (0.08, 4.01)  P=0.5749 | 0.33  (0.01, 60.16)  P=0.6748 | 2.62  (0.04, 190.05)  P=0.6593 | 1.31  (0.32, 5.31)  P=0.7049 |
| C versus A | 3.49  (1.07, 11.39)  P=0.0379 | 2.98  (0.22, 40.40)  P=0.4114 | 11.18  (0.30, 414.30)  P=0.1902 | 7.90  (2.81, 22.24)  *P=0.0001 |
| C versus B | 6.09  (1.12, 33.21)  P=0.0367 | 9.10  (0.07, 1112.59)  P=0.3678 | 4.27  (0.31, 59.36)  P=0.2802 | 6.03  (2.14, 16.97)  *P=0.0007 |

Note: To account for the multiplicity associated with pair-wise tests of rate ratios we employ a Bonferroni approach to inference. i.e. We modify our traditional α=0.05 level for declaring statistical significance to 0.0083 (i.e. the original 5% alpha-level divided by the number of pair-wise comparisons under consideration for a given model – here six).

| **Arm of Trial** | **Number Attending Outcome** | **Number Attending First ANC Outcome** | **Number Seeing Traditional Healer Outcome** | **Number Stating they Came for POBU Outcome** |
| --- | --- | --- | --- | --- |
| Intervention A versus Control | 1.77  (0.68, 4.58) P=0.242 | 1.24  (0.15, 10.07) P=0.840 | 0.81  (0.03, 19.27) P=0.899 | 32.56  (2.91, 364.15) P=0.005 |
| B versus Control | 2.91  (0.75, 11.25) P=0.121 | 1.17  (0.02, 59.41) P=0.938 | 6.13  (0.58, 64.88) P=0.131 | 122.68  (10.97, 1372.29) *P<0.0001 |
| C versus Control | 10.33  (5.74, 18.61) *P=<0.0001 | 6.19  (1.73, 22.14) P=0.005 | 15.24  (4.13, 56.27)  *P<0.0001 | 430.83  (46.56, 3986.37) *P<0.0001 |
| B versus A | 1.65 (0.36,7.63) P=0.523 | 0.94  (0.01, 68.85) p=0.978 | 7.54  (0.20, 291.15) P=0.279 | 3.77  (0.93, 15.19) P=0.062 |
| C versus A | 5.85 (2.31, 14.85) *P=0.0002 | 4.99  (0.58, 42.69) P=0.142 | 18.73  (0.86, 408.01) P=0.062 | 13.23  (4.72, 37.11) *P<0.0001 |
| C versus B | 3.55 (0.93, 13.51) P=0.063 | 5.30  (0.10, 277.10) P=0.409 | 2.48  (0.26, 23.48) P=0.427 | 3.51  (1.25, 9.85) *P=0.017 |

Rate ratios of primary and secondary outcomes between groups with delivery at healthcare facility used as denominator.

A = Intervention A

B = Intervention B

C = Intervention C

*statistically significant
